# Human Linguisticality and the Building Blocks of Languages

**DOI:** 10.3389/fpsyg.2019.03056

**Published:** 2020-01-31

**Authors:** Martin Haspelmath

**Affiliations:** ^1^Max Planck Institute for the Science of Human History, Jena, Germany; ^2^Department of British Studies, Leipzig University, Leipzig, Germany

**Keywords:** linguisticality, universal grammar, language faculty, convergent evolution, cultural evolution, natural kind entities

## Abstract

This paper discusses the widely held idea that the building blocks of languages (features, categories, and architectures) are part of an innate blueprint for Human Language, and notes that if one allows for convergent cultural evolution of grammatical structures, then much of the motivation for it disappears. I start by observing that human linguisticality (=the biological capacity for language) is uncontroversial, and that confusing terminology (“language faculty,” “universal grammar”) has often clouded the substantive issues in the past. I argue that like musicality and other biological capacities, linguisticality is best studied in a broadly comparative perspective. Comparing languages like other aspects of culture means that the comparisons are of the Greenbergian type, but many linguists have presupposed that the comparisons should be done as in chemistry, with the presupposition that the innate building blocks are also the material that individual grammars are made of. In actual fact, the structural uniqueness of languages (in lexicon, phonology, and morphosyntax) leads us to prefer a Greenbergian approach to comparison, which is also more in line with the Minimalist idea that there are very few domain-specific elements of the biological capacity for language.

## Introduction

This paper makes two interrelated claims and embeds them in ongoing discussions in linguistics and some adjacent areas:

(i) Humans’ biological capacity for language (=human linguisticality) is best studied from a broadly comparative perspective – comparing species, capacities, and languages.
(ii) The comparison of languages does not lead to immediate insights about human linguisticality, because languages have a very diverse range of building blocks whose similarities do not appear to be rooted in innate natural kinds.

That biolinguistics (=the study of the biological capacity for language) should adopt a broadly comparative perspective seems such an evident suggestion that it need not be mentioned, but *de facto*, the term “biolinguistics” has come to be associated with the ideas of a single scholar, Chomsky^1^, and much work in the Chomskyan tradition does not take a broadly comparative perspective. The vast majority of linguists working in the generative-grammar tradition consider only humans, only the capacity for language, and in addition, like most other linguists, they tend to focus on a single language.

Still, linguists who work on a single language tend to emphasize the broad implications of their work. In a recent introductory textbook on syntax, for example, the authors write that linguists are motivated by “the desire to understand the human brain.” (Koeneman and Zeijlstra, 2017: 3), even though their textbook talks almost exclusively about English syntax. Thus, here I emphasize the diversity of languages, and I note that their comparison is not at all straightforward. We cannot simply use the building blocks as established on the basis of Latin, English, or Chinese, and carry them over to all other languages. And even if we compare many different languages, it is not clear if our results contribute to “understanding the human brain” or other aspects of human biology.

This point is often underappreciated, even by many linguists who work on diverse languages. I conclude that biolinguistics must become much broader if it wants to go beyond speculation and gain lasting insights into the biological foundations of human language.

In the next section, I explain why I use the new term “linguisticality” for the human capacity for language, and how it relates to other widely used terms (“faculty of language,” “universal grammar”). Then I elaborate on the need for a broadly comparative perspective, before coming to the central point, the diversity of the structural building blocks of languages.

## Human Linguisticality, the “Language Faculty” and “Universal Grammar”

Linguisticality is the set of capacities that allows humans to learn and use languages in all their diverse forms (spoken, signed, written, vernacular, whispered, sacred, in song lyrics, in proverbs, in language games, and so on). Since linguisticality is a species-specific capacity and is invariant across the entire human population, it is appropriately studied from a biological perspective (in what might be called biolinguistic inquiry; but see n. 1).

The term *linguisticality*, introduced in this paper, was formed on the analogy of the term *musicality*^2^, which is used by musicologists to refer to the human capacity for music. For example, Honing (2018) says (see also Trehub, 2003):

“Over the years, it has become clear that all humans share a predisposition for music, just like we have for language. all humans, not just highly trained individuals, share a predisposition for music in the form of musicality – defined as a spontaneously developing set of traits based on and constrained by our cognitive abilities and their underlying biology.”

It may seem strange to propose a completely new term, linguisticality, for such a basic phenomenon, after hundreds of years of language study. And of course, many scholars have talked about linguisticality, but there is no other single term that could be used to make it clear exactly what is meant. Some authors talk about the “capacity for language” (as I did in (i) above), or the “language capacity” (e.g., Jackendoff, 1999), and these are certainly good alternative terms.

But many others simply use “language,” and this word is too vague. “Language” can refer to particular languages (sets of conventions used by particular speech communities), or to the use of a language in speech, or to the entire domain of phenomena related to language use and language systems. As an example of this vagueness, consider the expression “language evolution”: This can refer to the (biological) evolution of linguisticality, or to the (cultural) evolution (or diachronic change) of particular language systems. To be on the safe side, Jackendoff (1999) talks about “the evolution of the language capacity.” It would be clearer to distinguish between (biological) “evolution of linguisticality” and (cultural-diachronic) “evolution of languages”^3^.

The human capacity for language is in many ways like an instinct, and Pinker (1994) used “language instinct” as a book title. But much more common is another term: “language faculty.” This term seems to go back to Saussure’s (1916) *faculté du langage*, but nowadays, it is often used in a much narrower sense. While Rizzi (2004) continues the Saussurean tradition and uses it in the same sense as linguisticality^4^, many other authors use “language faculty” (or “faculty of language”) for a domain-specific cognitive module (sometimes called “the language organ,” Anderson and Lightfoot, 2002)^5^. For example, Chomsky et al. (2019) say at the beginning of their paper about the language faculty:

“Generative Grammar (GG) is the study of the linguistic capacity as a component of human cognition.”

If the language faculty is what generative grammarians study, then it must be the hypothesized domain-specific cognitive module, because generative grammarians do not (in practice) study domain-general aspects of human cognition and human auditory and articulatory abilities, which are also part of human linguisticality. This narrow understanding of the term “language faculty” was also used in 1978 in the famous “GLOW Manifesto” (by Jan Koster, Henk van Riemsdijk, and Jean-Roger Vergnaud):

“It appears quite likely that the system of mechanisms and principles put to work in the acquisition of the knowledge of language will turn out to be a highly specific “language faculty”.”^6^

And non-Chomskyan authors who find the evidence for a domain-specific module insufficient sometimes even say that they reject the language faculty, e.g.,

“the language faculty is, quite literally, empty: natural language emerges from general cognitive constraints, and. there is no innately specified special-purpose cognitive machinery devoted to language” (Christiansen and Chater, 2015: 1–2).

Christiansen and Chater do not, of course, reject the existence of human linguisticality – they would merely say that the human capacity for language consists of multiple different subcapacities that are not specialized for language, at least not for morphosyntax (they do accept that there may be a specialized capacity for speech processing; Lieberman, 1984).

In addition to this ambiguity of the term “language faculty” [referring to (i) linguisticality or (ii) to a hypothesized domain-specific cognitive module], additional confusion was created by Hauser et al. (2002), who introduced a distinction between “the faculty of language in the broad sense (FLB)” and “the faculty of language in the narrow sense (FLN).” The first, FLB, is the same as linguisticality^7^, but the second is much less clear (“FLN is the abstract linguistic computational system alone, independent of the other systems with which it interacts and interfaces”). The authors emphasize the special importance of recursion and suggest that “FLN only includes recursion,” which would mean that it is not domain-specific (see the discussion in Scholz et al., 2011: §2.3). Thus, FLN cannot be the same as the hypothesized domain-specific cognitive module (or language organ).

Finally, the term “universal grammar” (often abbreviated as UG)^8^ has often been used in this context by Chomskyans, but this is not an unambiguous term either. Most commonly, linguists use it for the set of building blocks (features, categories, and architectures) that they hypothesize to be innate:

“Universal grammar consists of a set of atomic grammatical categories and relations that are the building blocks of the particular grammars of all human languages, over which syntactic structures and constraints on those structures are defined. A universal grammar would suggest that all languages possess the same set of categories and relations.” (Barsky, 2016)

Chomskyan linguists rarely commit themselves to specifying exactly which categories they assume to be innate (see section “The Structural Uniqueness of the Building Blocks”)^9^, but the entire enterprise is built on these assumptions, because otherwise there would be no justification for using different criteria for different languages (cf. Croft, 2009). And at least for segmental features, there have been some very concrete proposals for UG features since the 1950s (the distinctive features of phonology, first proposed by Jakobson, Halle, and Chomsky). Moreover, there are many architectural proposals for the language system (e.g., the earlier distinction between deep structure and surface structure, or ideas about the ways in which phonology, syntax, and the lexicon interact), which are widely thought to be due to innate structures.

Since there is no doubt about the biological basis of human linguisticality, it is perfectly possible that not only the instinct to communicate, to imitate and to extract patterns from observed speech signals is innate, but that also a substantial number of specific structural building blocks (features, categories, and architectures) are in place before children start hearing their caretakers speak. The capacity for language would be like the capacity for taste, where culture-specific taste categories (which enable culture-specific recipes and cuisines to exist and to be transmitted) coexist with (and have an ultimate basis in) five innate basic taste categories (sweet, sour, salty, bitter, umami).

But in addition to this first (“innate categories”) sense of “universal grammar,” there is also a second sense, where UG is roughly synonymous with “domain-specific aspects of linguisticality”:

“No known ‘general learning mechanism’ can acquire a natural language solely on the basis of positive or negative evidence, and the prospects for finding any such domain-independent device seem rather dim. The difficulty of this problem leads to the hypothesis that whatever system is responsible must be biased or constrained in certain ways. Such constraints have historically been termed ‘innate dispositions,’ with those underlying language referred to as ‘universal grammar.’ Although these particular terms have been forcibly rejected by many researchers, and the nature of the particular constraints on human (or animal) learning mechanisms is currently unresolved, the existence of some such constraints cannot be seriously doubted.” (Hauser et al., 2002)

This formulation is much more careful and vague than the earlier quote from Barsky (2016). Hauser et al. (2002) apparently do not want to commit themselves to more specific claims here, but they still use the term “universal grammar.” In the above passage, they define UG as the domain-specific capacity to acquire a language, so if one doubts the existence of domain-specific components of linguisticality, one can say that there is a “UG hypothesis” (e.g., Da̧browska, 2015), and that one regards this hypothesis as “dead” (e.g., Tomasello, 2009). But there is also a third sense of UG, where it is the same as the “(broad) language faculty”^10^, and thus the same as linguisticality, e.g., in this quotation:

“The term Universal Grammar (UG) is simply a label for this striking difference in cognitive capacity between ‘us and them’ [=humans and non-human animals]. As such, UG is the research topic of GG: what is it, and how did it evolve in us?” (Chomsky et al., 2019).

Since there is no doubt about the difference in cognitive capacity between humans and non-humans, UG in this third sense is not a hypothesis^11^.

Thus, we have seen that the terms “language faculty” and “universal grammar” have been used in multiple and confusing senses in the literature. It is therefore best to use a new term, *linguisticality*, for the biological capacity for language, analogous to the term *musicality* for the biological capacity for music^12^. The term should not be taken as implying any further claims about the nature of this biological capacity. This should be taken as an empirical question.

## The Comparative Study of Linguisticality: Species, Capacities, Languages

In order to understand any biological behavioral trait or capacity (such as birdsong, or echolocation in bats, or web-building in spiders, or territoriality), it is important to study similarities across different species. This is a fundamental principle in all areas of behavioral biology, and it should of course also be adopted in biolinguistics. Concepts specific to human languages (such as *relative clause* or *determiner*) are unlikely to be useful for this kind of comparison. Some linguists have taken an interest in communicative or vocal behaviors of other animals, but they have more often emphasized the uniqueness of human languages (e.g., Anderson, 2004), and have not often looked broadly across species for similarities in order to understand how the various components of linguisticality might have arisen. What Fitch (2015) says about musicality applies in exactly the same way to the capacity for language:

“[The comparative principle] urges a biologically comparative approach, involving the study of behavioral capacities resembling or related to components of human musicality in a wide range of non-human animal species. This principle is of course a question familiar to most biologists, but remains contentious in musicology or psychology. ‘Broad’ in this context means that we should not limit our biological investigations to close relatives of humans (e.g., non-human primates) but should rather investigate any species exhibiting traits relevant to human musicality.” (Fitch, 2015: §2c)

For understandable reasons, many researchers have focused on comparing linguisticality in humans with the capacities of closely related species (especially chimpanzees and other primates, but also dogs), but as Fitch (2017) notes, “our understanding of cognitive evolution would be seriously incomplete if we focused exclusively on comparisons of humans with other primates (a narrow comparative approach). Fortunately, the genomic revolution has led to a widespread recognition of the fundamental conservatism of gene function in very disparate species. and there is a rising awareness that distant relatives like birds may have as much, or more, to tell us about the biology and evolution of human traits as comparisons with other primates.” I am not competent in this area, but it seems to me that Fitch is right that a biologically comparative approach is required for deeper understanding of linguisticality, just as such an approach is needed for any other biological trait of any species.

Second, we should also compare different capacities of humans if we want to understand each of them in a deeper way. Most linguists who claim to be interested in language as a cognitive capacity do not consider related capacities such as musicality, numerical cognition (e.g., Dehaene, 1997), visual perception. But just as we are unlikely to understand the behavioral capacities of a single species, we are unlikely to understand the biological bases of a single capacity in isolation. In view of the great specialization of the research fields, there are of course many practical impediments for such comparative research, but we should not delude ourselves and think that deeper insights will be possible without serious comparison across a range of behaviors. It is natural that most linguists work in those areas where they feel most comfortable, but the rhetoric of some linguists suggests that they expect (or have already reached) deep insights without any such comparison.

Third, and most importantly from my own perspective, we need to compare different languages in a serious way. I will elaborate on this in the next three sections, but here I will make two general points. First, it is of course true that Western linguists have considered different languages at least since the 17th century, when French and other European languages came into their view in addition to Latin. But this comparison became truly systematic and empirically serious only in the 19th century, and in that period, the comparison was historical. Many of the most influential philosophers and linguists of the 20th century that considered human language in general terms (e.g., Chomsky, 1965; Grice, 1975; Lyons, 1977; Langacker, 1987; Jackendoff, 2002; Goldberg, 2006) did not base their claims on a broadly comparative set of data. And second, within the Chomskyan community, a strongly aprioristic approach has always been dominant, even though since the 1990s, more and more linguists have tried to apply the mainstream generative grammar (MGG) formalisms to languages from outside Europe. The general direction of research has always been to show that languages other than English are really much like English after all (they have DPs/determiner phrases, configurational clause structure, standard word-class distinctions, a movement-based treatment of alternative orders, and so on). This is understandable, since all the textbooks are based on English, and the textbook assumptions are the only assumptions shared by all generative linguists. Thus, the desideratum of a biolinguistics that would be based on a broadly comparative approach without privileging any one language (like a biomusicology that does not, for example, privilege Western art music; Fitch, 2015; Honing et al., 2015) still needs to be fulfilled.

## How P-Linguistic Analyses May Illuminate Linguisticality: the Natural-Kinds Program

Instead of comparing languages in a systematic way, what the great majority of linguists (even those who emphasize their interest in larger questions) have been doing over the last few decades is engage in the study of particular languages. But how can analyses of particular languages (“p-linguistic analyses”) lead to insights into general questions about Human Language?

In Haspelmath (2020b), I observe that p-linguistics is not necessarily relevant to general linguistics (or “g-linguistics,” the study of Human Language), because the properties of individual languages are historically accidental. But there are two ways in which the study of a single language such as Mohawk (Baker, 1996) or French (Kayne, 1975) could contribute to our understanding of linguisticality: (i) We can study aspects of these languages which we know are not conventional, or (ii) we can study the conventional grammatical rules and hypothesize that they are based on innate building blocks (features, categories, and architectures). The first type would include psycholinguistic research (where speaker behavior is studied independently of speakers’ social knowledge) and stimulus poverty considerations.

Here I will focus on the second type of study: P-linguistic analyses that are based on the idea that all languages take their building blocks from a common innate blueprint or “framework” (see Haspelmath, 2010b for some discussion of this term). This approach has been very influential, and has often been presented as the only possibility for linguistics, even though it has always been clear that languages can also be studied as parochial systems of social conventions (because this is what we do when we take a language class). Let us look at a concrete example of a p-linguistic analysis.

Bloomfield (1933) observed that it is useful for English grammar to posit a special Determiner category that is unknown from Latin (and 19th century English grammar). As an approximation, we can say that English nominals consist of four slots, as in (1a). English Determiners include the forms in (1b).

(1)
  (a) Predeterminer – Determiner – Adjective – Noun
  (b) the, a(n), my, your, their, this, that

If we additionally say that the first three slots may be empty and that the two Predeterminers are *all* and *both*, we immediately explain why we can have all of (2a–e), but not, for example, (3a–c).

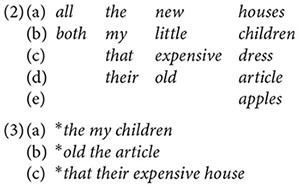

P-linguistic analyses consist in setting up categories of this kind and in specifying further conditions on the forms that can occur in the categorial slots (e.g., the English Determiner slot can be empty only if the noun is plural). So how could such an analysis illuminate not only the structure of English, but the biological capacity for language?

Bloomfield (1933) intended the Determiner category as a language-particular category for English, but it could of course be that it is an innate category, and that further categories such as those in (4) are likewise part of an innate blueprint. This is in fact what most syntacticians in the generative-grammar tradition claim, whether explicitly or (more commonly) implicitly.

(4) verb, noun, auxiliary, verb phrase, adposition, complementizer, case-marker, accusative, dative, ergative, agreement-marker, finite verb, reflexive, pronoun, coordinator, relative clause, singular, plural, first person, second, person, tense, mood, question word, question particle.

Clearly, the study of particular languages requires features and categories of this kind, and it also requires larger constructions (such as passive or causative constructions, or question-word constructions) and relations between constructions (of the kind that have been described by alternations or transformations). Again, one may hypothesize that the kinds of rules that one posits to express these regularities are part of human linguisticality from the very beginning (that they “belong to the language faculty,” as linguists often say).

As noted earlier, the idea that the building blocks of languages are innate is analogous to the finding that there are basic tastes that are genetically determined and do not vary across human populations, and one could also point to the idea that there are half a dozen basic emotions that are invariant and not subject to cross-cultural variation (cf. Barrett, 2006). The building blocks of languages would thus be natural kinds, like the building blocks of matter – the chemical elements.

Chemical elements are often said to be the best example of natural kinds, but biological species and their parts are also natural kinds in that they are given in advance by nature and are not identified by definitions. In order to identify gold (chemical symbol *Au*) or a red fox (*Vulpes vulpes*), we do not make use of definitions, but of a wide variety of symptoms (see Haspelmath, 2018 for further discussion of natural kinds in different disciplines). That the building blocks of languages are analogous to chemical elements has been argued extensively by Baker (2001). When serious chemical inquiry started in the 17th century, it was not clear whether all parts of the world (let alone the celestial bodies) consist of the same kinds of stuff. It was only through painstaking study of many particular substances from different parts of the earth (and also from meteorites, which were known to originate from outer space) that chemists eventually came to recognize that there are a few dozen elements of which all other substances are composed.

Thus, it is possible in principle that the study of the building blocks of particular languages gives us insight into the innate building blocks because the language-particular building blocks are actually drawn from the universal set. Determiner would not only be a category of English, but an element of the innate blueprint for Human Language (in other words, part of UG in the first sense, as in the Barsky quote in section “Human Linguisticality, the ‘Language Faculty’ and ‘Universal Grammar”’). This is what I call the natural-kinds program for finding the innate building blocks, making use of p-linguistic analyses.

## The Structural Uniqueness of the Building Blocks

The difficulty with the natural-kinds program is that different languages do not have the same building blocks. They show many similarities, and for most practical purposes, it is possible to translate from one language into another language. But there are also many differences which cannot be reduced to a set of elementary building blocks, at least at the present state of our knowledge.

For example, different languages carve up the same conceptual space in different ways, mapping to different word shapes. Where English has just a single word *hair*, French distiguishes between *cheveu* “head heair” and *poil* “beard or body or animal hair,” and Latin made a still different subdivision by distinguishing between *capillus* “heard or beard hair” and *pilus* “body or animal hair” (Koch, 2001: 1146). And where Russian distinguishes between *les* “forest or woods” and *derevo* “wood or tree,” French has *arbre* for “tree” and *bois* for “wood or woods or forest” (this example goes back to Hjelmslev’s discussion in the 1930s; cf. Haspelmath, 2003: 237). Ideally, this diversity of lexical semantics would be reduced to a small number of building blocks which combine to yield the diversity that we actually observe. And there is a proposal by Wierzbicka (e.g., Wierzbicka, 1996), to explain all word meanings on the basis of about 100 elementary (and presumably innate) semantic building blocks (“semantic primes,” or natural kinds). However, this research program has not been adopted by the discipline because Wierzbicka’s methodology for semantic decomposition does not seem rigorous. It seems that most linguists regard the goal as overambitious.

The situation is somewhat different in the case of phonological segments, where several proposals have been made for lists of innate building blocks that can be applied to all languages: The “distinctive features” for segments (first proposed by Jakobson et al., 1951 and made famous by Chomsky and Halle, 1968). However, while there are a number of authoritative proposals that are taught to students in textbooks (and can be looked up in encyclopedic articles)^13^, these still have the status of widely adopted proposals and do not have the status of generally accepted discoveries. Authors such as Blevins (2004) and Mielke (2008) have given good arguments for a different understanding of cross-linguistic similarities in phonology, where each language is analyzed in its own parochial terms and cross-linguistic similarities derive from diachronic (“evolutionary”) or adaptive tendencies. And authors like Lass (1984) and Simpson (1999) have pointed out that comparing phoneme inventories across hundreds of languages (as is done by Maddieson, 1984 and others) is not possible, because a phoneme system is determined by language-particular generalizations. Even if the distinctive features were universal, the organization of phoneme inventories is unique in every language. It was noted by Trubetzkoy (1939), in the founding document of modern phonology, that the French /t/ and the Greek /t/ are not the same element because they occur in different contrasts in their respective systems – they are structurally unique elements that we happen to use the same notation for. Phonological research over the last 80 years has not led to any different conclusions. Even though there are many obvious similarities, each language has its own system (and its own building blocks), and we do not know how to reduce these systems to a set of innate natural kinds.

In the case of syntactic building blocks, the situation is still different from lexical semantics and phonology, but not better, despite Baker’s (2001) suggestion that comparative syntactic work has advanced as much as comparative chemical work in the mid 19th century, and that our Mendeleev could just be around the corner, providing syntacticians with a “periodic table of innate syntactic elements” to be taught in syntax classes and to be used in linguistic analysis. But in practice, this is not the case. The fate of Bloomfield’s “determiner” concept is symptomatic in this regard. In the late 1980s, it was proposed that the “Determiner” plays a more important role in English syntax than was previously thought, and as soon as it got more prominent in English syntax papers, the concept was transferred to other languages where it cannot be motivated in the same way (on the assumption that it is not a unique building block of English, but must reflect the innate blueprint). For example, in Modern Greek, the definite article and the demonstrative co-occur and thus cannot be in the same slot (e.g., *aftó to spíti* [that the house] “that house”). Different criteria were used in different languages for a determiner, and it was simply assumed that all languages have it, even when it is not overt most of the time. The motivation for assuming such an innate category came from English, not from comparative studies^14^.

The general situation in syntax is different from lexical semantics in that many syntacticians assume that there is a fixed list of innate building blocks (whereas few lexical semanticists assume this), but unlike phonologists, syntacticians have not come up with an authoritative proposal. The different “frameworks” that arose in the 1980s have proposed very different sets of basic building blocks (e.g., Relational Grammar, Blake, 1990; Lexical-Functional Grammar, Bresnan, 2001; Mainstream Generative Grammar; Adger, 2003), and within the numerically dominant MGG school, there are many different views which are often mutually incompatible. Authors like Cinque (1999) have argued for a “cartographic” approach in which many dozens of innate categories are proposed, but other authors, inspired by philosophical “Minimalism,” have argued that it is quite impossible for so many natural-kind categories to be innate because they could not have evolved (this has been called “Darwin’s problem”, e.g., Bolhuis et al., 2014: 5). And finally, actually practicing language describers have not found any use for any of these proposals. Unlike the proposals of phonological theorists, which have sometimes been made use of in comprehensive grammatical descriptions, the “framework-based” proposals play no role in the training for linguistic fieldwork (cf. Payne, 1997; Chelliah and De Reuse, 2011).

Language describers basically still follow Boas’s (1911) exhortation to describe each language in its own terms (just as anthropologists describe each culture as a unique set of practices), rather than imposing some preconceived scheme on them, even though they have realized that comparative work can help them because of the many similarities of languages^15^.

Now one may of course object to this negative assessment by observing that our current lack of a complete theory of innate building blocks does not mean that such a theory is impossible. This is true, but there seems to be little awareness among linguists who are pursuing this program that a natural-kinds theory is not necessary, and that much of the current research is based on the unquestioned presupposition that there is no alternative to it. The next section will sketch such an alternative: The idea that the cross-linguistic convergence of linguistic features (leading to striking similarities between languages) may be due to convergent cultural evolution, rather than to innate natural kinds.

## A Biological Blueprint Vs. Convergent Cultural Evolution

In various domains of study, similarities across different phenomena may have quite different causes, and it may be challenging to identify them. For example, biologists are not sure whether the similarities between species in different taxa (e.g., wings in birds and bats) can be exclusively explained by convergent biological evolution. Alternatively, one might think that many of the similarities are due to constraints on basic structures that cannot be overridden by biological adaptation. These do not seem to be currently well-understood, but at least one biologist, Stephen Jay Gould (1941–2002) became famous for suggesting that the power of convergent evolution has been overestimated (Losos, 2017 provides some very accessible recent discussion).

Similarly, the explanation for the similarities between languages may not lie exclusively in convergent cultural evolution. There may be specific biological constraints on possible language systems, just as there are (apparently) specific biological constraints on taste categories and emotion categories. These are not currently well-understood by linguists (as noted earlier), but they may well exist, just as there may be “constraints on basic structures” in biology.

However, it should be self-evident that there are also many similarities between languages that are sufficiently explained by convergent cultural evolution. Just as nobody doubts that the cross-cultural existence of similar kinds of houses, tools, weapons, musical instruments and governance structures (e.g., chiefdoms) is not due to a genetic blueprint for culture but to convergent cultural evolution, there is also no real doubt that many similarities in the words of languages are due to cultural similarities and need no biological explanation. For example, many languages in the 21st century have short words for mobile phones, and these can be created in different ways (by abbreviating longer terms, e.g., Polish *komórka* from *telefon komórkowy*, by using a brand name, e.g., *Natel* in earlier Swiss German, or even letter abbreviations like *HP* in Indonesian, for *hand phone*). Nobody would doubt the claim that this is an adaptive feature of these languages that is not due to anything in our biology.

It is an obvious feature of human linguisticality that human groups form linguistic conventions that are subject to change. The change is not fast, and speakers of the same community usually find it easy to understand each other even across three or four generations. But over the centuries, it accumulates, and when cultural change is fast (as with mobile phones and many other terms for modern technology), languages may change fast to adapt to the speakers’ needs. Thus, languages are subject to cultural evolution (Croft, 2000a; Mesoudi, 2011), and when there are selective pressures, this change may be adaptive. Many general aspects of languages are apparently due to the adaptation of language structures to the needs of the speakers. Not only the length of words can be explained as an adaptation to their predictability and frequency (as in the mobile phone example; cf. Zipf, 1935; Kanwal et al., 2017), but also the length and presence of grammatical markers (see Haspelmath, 2020a on asymmetric coding in grammar). In phonological systems, not only vowel dispersion, but also the structure of consonant inventories is clearly adaptive (e.g., Flemming, 2017). And in morphosyntax, not only asymmetric coding tendencies, but also many word and clause ordering tendencies can be explained on the basis of general processing preferences that are not specific to linguisticality (Hawkins, 2014). Similarities across languages in terms of word class categories (Croft, 2000b) and reflexive constructions (Haspelmath, 2008) have likewise been explained in functional-adaptive terms. Basically, all of the categories listed in (4) above may well be similar across languages because they serve universal needs of speakers.

Thus, linguists who compare languages and want to explain patterns that are general across languages and cannot be due to historical accidents need to consider two possible sources of these similarities:

(i) convergent cultural evolution of languages to the same needs of speakers,
(ii) constraints on biologically possible language systems: innate building blocks (natural kinds) that provide a rigid blueprint for languages.

The two answers might even be correct simultaneously, but there is of course also a question of instrinsic likelihood: How likely is it that a grammatical feature is part of an innate blueprint, which would have had to evolve biologically within a million years or less (“Darwin’s problem”)? By contrast, how likely it is that an adaptive feature of a language system would have evolved culturally over a few generations, given that we observe such changes wherever we look in the historical record?

## Conclusion: the Building Blocks of Languages Under a Mimimalist Lens

If we take a comparative approach to human linguisticality, we observe at the species level that linguisticality is unique to humans. But at the level of different human communities, we observe that each language is unique, just as other aspects of human cultures are unique to each culture. Languages exhibit many similarities, but just as biological similarities need not be due to genetic identity, linguistic universals need not be due to an innate blueprint. Analogously to biological convergent evolution, which can produce similar outcomes in unrelated taxa (eyes in insects and vertebrates), the similarities between languages may be due to convergent cultural evolution. This means that the description and comparison of languages does not lead to immediate insights into human linguisticality (see (ii) in §6).

As noted, an alternative possibility is that some of the linguistic universals are due to a biological blueprint [a “universal toolkit,” as Jackendoff (2002: 75) calls it], and sometimes a biological and a cultural-evolution explanation may be simultaneously appropriate. Linguists have found it very difficult to decide between these two possibilities, but a “minimalist lens” would seem to suggest that as little as possible should be attributed to biological constraints (i.e., to natural-kind categories). There are some evident biological constraints in other parts of cognition, so it cannot be ruled out that categories like “noun” and “verb,” or “consonant” and “vowel,” or even “deep structure” and “surface structure,” are innate building blocks of our cognition in the same manner as the five basic tastes^16^.

But general principles of explanatory economy (cf. the “cost scale” of explanatory factors in Haspelmath, 2019: 16) would suggest that one should posit innate building blocks of languages only if convergent-evolution explanations do not exist or are very unlikely. As far as I can see, the evidence from comparative linguistics does not currently provide strong evidence for innate building blocks of grammars^17^. While my perspective is shaped by the “functionalist” tradition of comparative linguistics (Greenberg, 1963; Croft, 2003; Givón, 2010), this provides an interesting convergence with some Chomskyan Minimalists such as Hornstein (2018), who recognize that there may be far fewer innate building blocks that than were often assumed in the past^18^.

Nevertheless, we need to pursue all avenues in order to come to a better understanding of human languages and of human linguisticality. I do not dismiss the natural-kinds program, and linguists who pursue the natural-kinds program cannot dismiss the successes of the convergent-evolution approach^19^.

## Author Contributions

MH conceived and wrote the manuscript.

## Conflict of Interest

The author declares that the research was conducted in the absence of any commercial or financial relationships that could be construed as a potential conflict of interest.

## Footnotes

1 For example, the “Biolinguistics Manifesto” (Boeckx and Grohmann, 2007) mentions Chomsky’s name in the first line, and seven times in the first paragraph (see also Di Sciullo and Aguero-Bautista, 2016, where biolinguistics is likewise closely linked to the Chomskyan approach, as well as Martins and Boeckx, 2016a, b: §2.6). Since Chomsky’s ideas are highly contentious and polarizing, many linguists will not want to be associated with the term *biolinguistics*, even though it is in principle neutral and transparent (like *biomusicology*, *bioacoustics*, *biomechanics*, etc.).

2 The analogy is *music/musical/musicality* = *language/linguistic/linguisticality*. To be sure, the term *musical* not only means “music-related” (just as *linguistic* means “language-related”), but also “capable of making/enjoying music,” whereas *linguistic* does not have this sense (presumably because every human is “linguistic”; though infants are often called *prelinguistic*). The term *linguisticality* is thus not completely transparent. (It should be noted here that “linguistic” is also sometimes used as an adjective for *linguistics*, the discipline of language study. Linguisticality should of course be understood in the first sense. Thus, in a language like German, which distinguishes between *sprachlich* “language-related” and *linguistisch* “linguistics-related,” the counterpart of *linguisticality* is *Sprachlichkeit*.)

3 Often, such vague terminology does not do any harm because the context makes it clear what is intended, but in this particular case, there is a serious problem – so much so that it is unclear what is in the scope of the *Journal of Language Evolution* (biological evolution, or cultural evolution, or both?). In response to a critical blogpost of mine (https://dlc.hypotheses.org/894), the editors changed the wording of the Aims and Scope statement, but it is still not very clear.

4 “[The fundamental object of inquiry is] “the language faculty,” the “instinctive tendency” for language, according to the terminologies used by Ferdinand de Saussure and Charles Darwin, respectively: a cognitive capacity rooted in the biological endowment of our species which allows us to acquire the natural language(s) we are exposed to in childhood and use it for communication, social interaction, and the expression of thought” (Rizzi, 2004: 323).

5 Compare also the following quotation: “The faculty of language can reasonably be regarded as a “language organ” in the sense in which scientists speak of the visual system, or immune system, or circulatory system, as organs of the body” (Chomsky, 2000: 4).

6 https://glowlinguistics.org/about/history/manifesto/

7 “We take as uncontroversial the existence of some biological capacity of humans that allows us (and not, for example, chimpanzees) to readily master any human language without explicit instruction” (Hauser et al., 2002: 1571).

8 Sometimes the upper-case spelling “Universal Grammar” is used, maybe to match the abbreviation (UG), or maybe to make it clear that this is an opaque term that is not meant to refer to a “grammar” that is “universal.”

9 With some exasperation, but not without justification, Tomasello observes: “Ask yourself: what exactly is in universal grammar? Oh, you don’t know – but you are sure that the experts (generative linguists) do. Wrong; they don’t. And not only that, they have no method for finding out.” (Tomasello, 2009; see also text footnote 14 below on the last point).

10 Hornstein (2019) basically equates them and consistently uses the term “FL/UG”. He says: “If we call this meta-capacity [to acquire a grammar] the *Faculty of Language* (FL), then humans necessarily have an FL and necessarily have UG, as the latter is just a description of FL’s properties.” (Hornstein, 2019: 189).

11 The second or third sense of the term UG is also used by Pinker (2007): “This idea [=universal grammar] sounds more controversial than it is (or at least more controversial than it should be) because the logic of induction mandates that children make *some* assumptions about how language works in order for them to succeed at learning a language at all. The only real controversy is what these assumptions consist of: a blueprint for a specific kind of rule system, a set of abstract principles, or a mechanism for finding simple patterns (which might also be used in learning things other than language).” – As Pinker notes, the first assumption, that what is innate is “a blueprint” (=a set of innate categories and architectures), is indeed controversial, but this is what most generative linguists who study languages have been assuming. And it is this “UG of innate categories,” or “UG of natural kinds,” that I will discuss further below.

12 Fitch (2015: §1) says about musicality and biomusicology: “Human MUSICALITY refers to the set of capacities and proclivities that allows our species to generate and enjoy music in all of its diverse forms. A core tenet of bio-musicology is that musicality is deeply rooted in human biology, in a form that is typical of our species and broadly shared by members of all human cultures.” The same could be said analogously about linguisticality and biolinguistics.

13 e.g., https://en.wikipedia.org/wiki/Distinctive_feature

14 Thirty years after the original proposal, many authors still work with a universal determiner category, though many others have raised objections. As a recent workshop on the topic NP vs. DP (at the Annual Meeting of the DGfS, Bremen, 2019) showed, there is no agreement on methodological standards for determining whether such a universal building block exists.

15 For example, Epps (2011: 648) says that fieldworkers should “produce descriptions in formats that will enable and facilitate comparison across languages, but also remain true to the languages themselves, without forcing them into ill-fitting predetermined categories (Gil, 2001; Haspelmath, 2007, 2010a).”

16 Recall from section “Human Linguisticality, the ‘Language Faculty’ and ‘Universal Grammar”’ that the term “universal grammar” has not only been used in the concrete sense of specific innate building blocks (which can be investigated by a natural-kinds program as currently carried out by many syntacticians), but also in the sense of “whatever is domain-specific to human linguisticality.” This is not something that the comparative study of languages can contribute to, but it seems quite likely that other considerations lead one to assume such domain-specific capacities (e.g., in speech perception and word learning, as noted by Pinker and Jackendoff, 2005: §3).

17 This would also explain why the natural-kinds program (section “How P-Linguistic Analyses May Illuminate Linguisticality: The Natural-Kinds Program”) has not been very successful so far (as noted in section “The Structural Uniqueness of the Building Blocks”).

18 However, Hornstein makes a distinction between substantive and structural universals (following Chomsky, 1965), and he is still quite optimistic about the latter being innate [“the Subjacency Principle, Principles of Binding, Cross Over effects, X’ theory with its heads, complements and specifiers; these are all structural notions that describe (and delimit) how Gs function”]. But I do not think that the phenomena described by these terms are any different from the asymmetric coding, word-class and word order universals that have been successfully explained in functional-adaptive terms by Croft, Hawkins, and Haspelmath.

19 Pinker and Jackendoff (2005) say that it “seems likely” that constituent structure, word order, agreement and case are specific to language and they simply assume that they are biological. For some reason, they do not even consider the possibility that the corresponding phenomena in languages are due to convergent cultural evolution (even though short markers of semantic roles, as provided by case flags and agreement markers, are just as useful for all speakers as short word for mobile phones; see Lehmann, 1982 for a functional account of agreement phenomena).
